# Hydrogen Bonding in Graphene

**DOI:** 10.1002/smll.73733

**Published:** 2026-05-22

**Authors:** Norbert H. Nickel, Jörg Rappich, Karsten Hinrichs, Tilmann J. Neubert

**Affiliations:** ^1^ Helmholtz‐Zentrum Berlin für Materialien und Energie GmbH Nanoscale Solid‐Liquid Interfaces Berlin Germany; ^2^ Friedrich‐Schiller‐Universität Jena Institut für Technische Chemie und Umweltchemie Philosophenweg 7a Jena Germany

**Keywords:** graphene, hydrogen concentration, hydrogen density‐of‐states

## Abstract

Effusion measurements are performed to determine the hydrogen concentration and its binding energies in large area graphene. The samples were grown by chemical vapor deposition using deuterium and deuterated methane as readily identifiable isotopes that duplicate hydrogen chemistry. In a second step, graphene is transferred to silicon substrates covered with an oxide layer of different thickness. H binding energies and the H density‐of‐states distributions are derived from the effusion spectra. The data show a well defined peak in the H density‐of‐states at 1.97 eV that is influenced by the thickness of the oxide layer. This is discussed in terms of a model that describes the dissociation of C−H bonds.

## Graphical abstract

The hydrogen concentration and binding energies in large area graphene are determined from effusion measurements. The samples are grown using chemical vapor deposition using deuterated methane and deuterium as readily identifiable isotopes. Total hydrogen concentrations of 1.2 ‐ 6.9 × 10^13^ cm^−2^ are obtained. The hydrogen density‐of‐states reveals an effective binding energy of 1.97 eV.

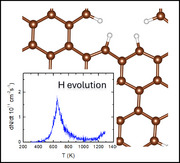

## 1 Introduction

The interaction of hydrogen with graphene has been investigated in great detail previously. The properties of hydrogen comprise chemisorption on graphene, which can change the hybridization of the carbon atoms from ${\mathrm{sp}}^{2}$ to ${\mathrm{sp}}^{3}$ [1]. An exposure of graphene at low temperatures was proposed for the fabrication of graphene/graphane patterns [2]. On the other hand, when the hydrogen coverage is low H atoms cluster on the graphene surface forming short dimers and ellipsoids. According to scanning tunneling microscopy and ab inito calculations, these structures were assigned to ortho and para hydrogen molecules [3]. For the growth of large area graphene, the ability of hydrogen to act as an etching reagent is important, since it regulates domain sizes during chemical vapor deposition (CVD) [4, 5]. Besides the use for electronic devices [6] graphene can be used for isotope separation of hydrogen, deuterium, and tritium using electrochemical pumping [7].

Because of the large surface area relative to its volume and the ability to tune the chemical properties of graphene, it has come into focus for hydrogen storage [8, 9, 10]. For graphene nano sheets doped with Ni and Al a hydrogen storage capacity of 5.7 wt.% was reported [11]. On the other hand, ab‐initio calculation performed on Ti decorated iridia‐graphene predicts a storage capacity of up to 7.7 wt.% [12]. Most of these reports have in common that graphene is modified with metal ions to allow for the storage of hydrogen. Therefore, the question arises whether pristine undoped graphene can be used to store hydrogen.

For numerous other semiconductors, including ZnO and single crystal silicon it has been shown that the growth and fabrication steps give rise to the incorporation of hydrogen atoms. This process is driven by thermodynamics [13]. Hence, graphene grown by chemical vapor deposition (CVD) is expected to contain hydrogen. In this publication, experimental data on hydrogen concentration and bonding in CVD‐grown graphene are presented. The data were obtained from effusion measurements and are presented as hydrogen density‐of‐states distributions.

## 2 Experimental Details

Large area graphene was grown on copper foil using the catalytic decomposition of methane (${\mathrm{CH}}_{4}$) in a chemical vapor deposition process. In a first step, the 25 µm thick Cu foil was cleaned in an ultrasonic bath of isopropyl and acetone. Then, the surface oxide layer was etched off using acetic acid. Prior to the growth of graphene, the Cu substrates were exposed to hydrogen at elevated temperatures to ensure the removal of residual oxygen atoms from the Cu surface. The growth of large area graphene was performed at a temperature of 1050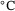 by adding methane to the hydrogen gas flow. To increase the sensitivity of gas effusion measurements, deuterium ($D_{2}$) and deuterated methane (${\mathrm{CD}}_{4}$) were used for the graphene growth. Deuterium is readily identifiable and duplicates hydrogen chemistry. Therefore, the terms hydrogen and deuterium will be used interchangeably in the following discussion. More details on CVD growth of graphene are given in a paper by Zheng et al. [14].

In a second step, the graphene layers were transferred to crystalline silicon substrates. Prior to the transfer, the native oxide was removed using HF, and a thermal ${\mathrm{SiO}}_{2}$ layer was grown. The thickness of the ${\mathrm{SiO}}_{2}$ layer varied between 0 and 200 nm. For the transfer process, the graphene layer was protected with nitrocellulose. The Cu foil was etched off with concentrated ${\mathrm{FeCl}}_{3}$, and the graphene/nitrocellulose stack was washed successively with dilute hydrochloric acid and deionized water. After the transfer step, the samples were dried for 3 h at 60 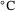. Finally, the protective nitrocellulose coating was carefully dissolved with ethyl acetate. Prior to and after the transfer the quality of the graphene layers was monitored using Raman scattering. The excitation wavelength was 632.8 nm. The morphology of the transferred graphene layers was characterized using atomic force microscopy (AFM).

Information on hydrogen concentration and bonding were obtained from gas effusion measurements. Samples with a size of 1.0 × 4.0 ${\mathrm{cm}}^{2}$ were heated with a heating rate of 20 K $\min^{-1}$ in ultra‐high vacuum. During the heating cycle the flux of $H_{2}$, HD, $D_{2}$, and ${\mathrm{CD}}_{4}$ were measured with a quadrupole mass spectrometer. Using the known neon flux through a capillary, the relative ion currents were calibrated to absolute values.

To evaluate potential paths for the dissociation of C−H bonds at the graphene sheets ab initio calculations were performed using density functional theory. Employing the Vienna Ab‐initio Simulation Package [15, 16] the generalized gradient approximation (GGA) was used according to Perdew, Burke, and Ernzerhof [17]. The supercell contained a total of 42 atoms with a vacuum region of 12.3 Å.

## 3 Raman Scattering

**Figure 1:**
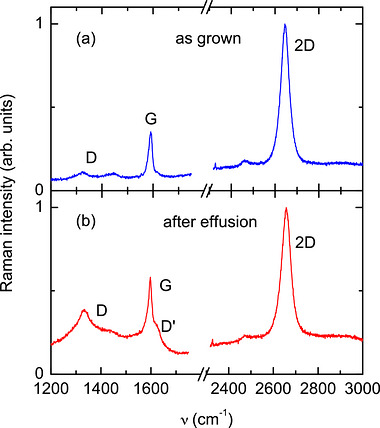
Raman backscattering spectra of CVD grown graphene normalized to the 2D phonon mode. (a) shows the phonon modes of CVD grown graphene transferred to a silicon substrate that was coated with 200 nm ${\mathrm{SiO}}_{2}$. Phonon modes obtained after the gas effusion measurement are shown in (b). The Raman measurements were performed at room temperature using an excitation wavelength of 632.8 nm.

A typical Raman spectrum of CVD graphene obtained after transfer to a crystalline silicon substrate coated with a 200 nm thick oxide layer is shown in Figure 1a. The D, G, and 2D modes are located at ≈1324, ≈1591, and ≈2646 ${\mathrm{cm}}^{-1}$, respectively. The presence of a small D peak with a relative intensity of 0.1 and an intensity ratio of $I_{D}/I_{G}\approx$ 0.3 and $I_{2D}/I_{G}\approx$ 2.86 are indicating that the CVD‐grown graphene is single layer graphene with a very low degree of disorder and defects.

**Figure 2:**
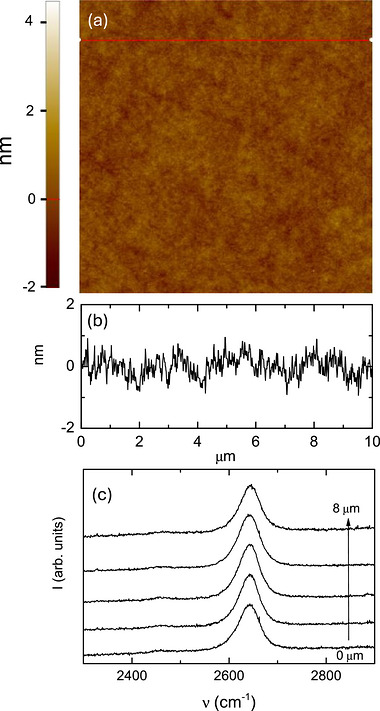
Atomic force microscopy micrograph of a 10 × 10 µm^2^ area of graphene on ${\mathrm{SiO}}_{2}$ (a). The surface roughness measured along the red line is shown in (b). In (c) Raman backscattering spectra of the 2D phonon mode are shown. The spectra were taken along an 8 µm long line.

The morphology of the graphene layers was characterized using AFM. An AFM micrograph is shown in Figure 2a. The scanned area indicates a homogeneous graphene layer. To gain further insight into the morphology and to determine the number of graphene layers, Raman line scans were performed. Figure 2c shows the 2D phonon mode of graphene taken at various positions along an 8 µm line. The shape of the 2D phonon mode and the peak position are excellent indicators of whether single‐ or multi‐layer graphene is present [18, 19, 20]. All Raman spectra taken along the 8 µm line show a 2D phonon mode with a Lorentz line shape at a wavenumber of ≈2645 ${\mathrm{cm}}^{-1}$. This clearly shows that the investigated specimens consist of single‐layer graphene.

In a gas effusion experiment the specimen is heated up to a temperature of 1000 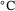. The question arises whether the effusion measurement alters or damages the graphene layers. Therefore, Raman spectra of the graphene layers were taken after the effusion measurement. Figure 1b shows the phonon spectrum of graphene on a 200 nm thick ${\mathrm{SiO}}_{2}$ layer after the effusion measurement. The 2D mode shifted by 9 ${\mathrm{cm}}^{-1}$ to a higher wave number of ≈2657 ${\mathrm{cm}}^{-1}$, which is indicative of compressive strain [21]. Furthermore, the intensity ratios of $I_{D}/I_{G}$ and $I_{2D}/I_{G}$ changed to ≈ 0.67 and 1.72, respectively, indicating that the quality of the graphene sheet is decreased. This is accompanied by a pronounced increase in the intensity of the D mode to a relative value of 0.39, suggesting that the effusion measurement results in the formation of defects. In addition, the D' band is visible after the effusion measurement. The observation of the D' band has also been attributed to the presence of defects. Depending on the magnitude of the intensity ratio $I_{D}/I_{D^{\prime }}$ different defect structures have been assigned. It has been suggested that *I*_D_/*I*_D'_ ≈ 13, ≈ 7, and ≈ 3.5 indicate the presence of ${\mathrm{sp}}^{3}$ hybridization, vacancies, and defects related to grain boundaries, respectively [22]. After the effusion measurement a ratio of $I_{D}/I_{D^{\prime }}\approx 1.4$ is obtained. This suggests that the newly formed defects are related to grain‐boundaries, which are common in large area CVD graphene [23].

## 4 Effusion Measurements

**Figure 3:**
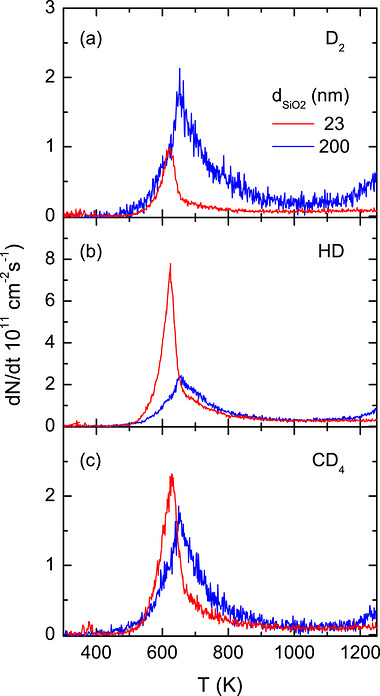
Effusion rate, dN/dt, as a function of temperature. The data obtained for molecular HD, $D_{2}$, and ${\mathrm{CD}}_{4}$ are shown in (a), (b) and (c) respectively. The data depicted by the red and blue curves were taken from large area graphene on ${\mathrm{SiO}}_{2}$ with a thickness of 23 and 200 nm, respectively. The measurements were performed with a heating rate of 20 K min^−1^.

In Figure 3, the effusion rates, dN/dt, for different hydrogen containing molecules are shown as a function of temperature. The molecular deuterium flux is shown in Figure 3a. For graphene on 23 nm thick ${\mathrm{SiO}}_{2}$ the $D_{2}$ flux peaks at a temperature of 624 K. However, when the thickness of the ${\mathrm{SiO}}_{2}$ layer increases to 200 nm the maximum $D_{2}$ flux shifts to a higher temperature of 655 K. Integration of the effusion spectra shows that the deuterium concentration for the graphene layer transferred to the 200 nm thick ${\mathrm{SiO}}_{2}$ layer is higher and amounts to about 6.9 × 10^13^ cm^−3^. The same temperature shift of the effusion peak is observed for HD and ${\mathrm{CD}}_{4}$ molecules (see Figure 3b,c). However, the maximum flux of HD and ${\mathrm{CD}}_{4}$ decreases with increasing oxide thickness.

Since graphene was grown with purely deuterated gases, the question about the origin of HD molecules arises. One possible source of hydrogen is residues from the transfer process. However, if that were the case, a dependence of the HD effusion peak on the ${\mathrm{SiO}}_{2}$ thickness should not be observed, since the residues would always be on the graphene side of the samples. A more plausible explanation is that hydrogen originates from the crystalline silicon (c‐Si) substrates underneath the ${\mathrm{SiO}}_{2}$ layers. c‐Si contains a considerable amount of hydrogen. Recently, it has been shown that c‐Si grown by the float‐zone technique contains a hydrogen concentration of about 9.2 × 10^17^ cm^−3^ [13]. During the effusion experiment H atoms migrate from the bulk of the substrate to the surface. At the graphene surface these H atoms recombine with D atoms from the graphene layer forming HD molecules. This is a strong indication that the D atoms in the graphene layers are atomically bound.

**Figure 4:**
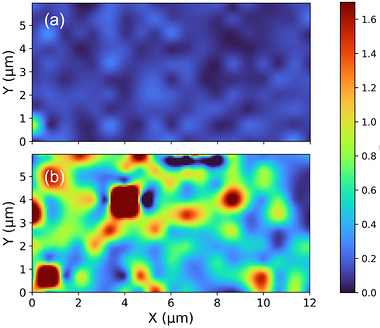
Raman maps of graphene showing the intensity ratio $I_{D}/I_{G}$ measured before (a) and after (b) the effusion experiment.

Interestingly, the effusion data also show a peak for ${\mathrm{CD}}_{4}$ at the same temperatures as HD and $D_{2}$. Most likely, some hydrocarbons form from weakly bound terminal C atoms that may be present at point defects and grain boundaries. To support this, Raman mapping of pristine graphene and graphene after an effusion measurement were measured. Figure 4a shows the intensity ratio of the D to G phonon modes for pristine graphene. After gas effusion, the intensity ratio $I_{D}/I_{G}$ increases indicating an increase in the defect concentration (b). This is consistent with the data shown in Figure 1. Hence, it is conceivable that C atoms originate from these sites. At the hot filament of the quadrupole mass spectrometer hydrocarbons can recombine to methane [24]. This secondary reaction path does not indicate a significant degradation of the graphene layer, which is consistent with a comparably small increase of the defect‐related phonon modes (see Figure 1). Moreover, the majority of the graphene bulk remains pristine even after heating to 1000 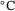, which is consistent with its high thermal conductivity of about 5 kW/mK [25] and reports on thermal stability of up to 2373 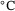 [26].

In contrast to molecular deuterium, for HD the effusion rate is higher when graphene is transferred onto a substrate with a thinner ${\mathrm{SiO}}_{2}$ layer. Although hydrogen diffusion in ${\mathrm{SiO}}_{2}$ is fast [27, 28] the difference in thickness by about a factor of ten has a significant influence on the number of H atoms that arrive from the c‐Si substrate at the graphene layer at any given time. Hence, the formation of HD and its effusion rate depend on the oxide thickness.

The effusion rate for ${\mathrm{CD}}_{4}$ changes by only 24%. This might be due to D atoms first being injected into the oxide layer prior to diffusing back to the graphene surface where ${\mathrm{CD}}_{4}$ is formed. For thicker ${\mathrm{SiO}}_{2}$ the number of D atoms that are diffusing back to the graphene surface decreases, resulting in a lower formation rate of ${\mathrm{CD}}_{4}$.

From the effusion data the total deuterium concentration can be determined by integrating the individual spectra for HD, $D_{2}$, and ${\mathrm{CD}}_{4}$ and taking the heating rate and sample geometry into account. The obtained D concentration varies from sample to sample but is consistently found in the range of $C_{D}$ = $10^{13}$ to $10^{14}$ ${\mathrm{cm}}^{-2}$ (see Table 1).

**Table 1:** Deuterium concentration, $C_{D}$, measured for graphene on c‐Si substrates with different oxide thickness, $d_{SiO_{2}}$.

$d_{SiO_{2}}\left(\mathrm{nm}\right)$	$C_{D}$ (${\mathrm{cm}}^{-2})$
0	1.9 × 10^13^
12	1.2 × 10^13^
23	6.4 × 10^13^
95	1.7 × 10^13^
200	6.9 × 10^13^

**Figure 5:**
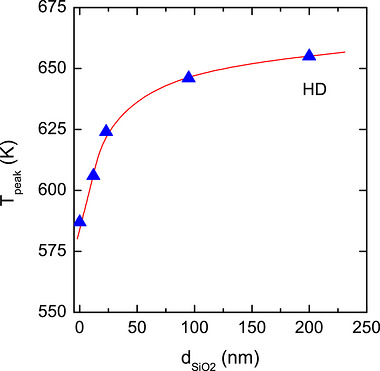
Temperature of the maximum effusion rate, $T_{peak}$, for HD molecules as a function of the oxide layer thickness, $d_{SiO_{2}}$.

The data depicted in Figure 3 show that the maximum molecular flux, max(dN/dt), for HD, $D_{2}$, and ${\mathrm{CD}}_{4}$ occurs at the same temperature, $T_{peak}$, for a given oxide layer thickness. Therefore, it is sufficient to plot $T_{peak}$ for only one molecule as a function of the oxide thickness. In Figure 5, $T_{peak}$ is plotted as a function of the oxide‐layer thickness, $d_{SiO_{2}}$. For samples that do not contain an oxide layer between c‐Si and graphene dN/dt peaks at a temperature of $T_{peak}$ = 587 K. With increasing oxide thickness the maximum of dN/dt shifts to a temperature of $T_{peak}$ = 655 K for $d_{SiO_{2}}$ = 200 nm. This, however, is a surprising observation since the deuterium binding energy in graphene should not depend on the oxide layer thickness.

## 5 Hydrogen Binding Energy

The effusion spectra can be further analyzed to gain insight into parameters such as the hydrogen chemical potential and the H density‐of‐states. In general, the hydrogen concentration is obtained by integrating over the H density‐of‐states, N(E), and taking into account the occupation function according to

(1) $$C_{D}=\int N_{D}\left(E\right)f\left(E,\mu_{D},T\right)dE$$

here, E represents the hydrogen binding energy, $\mu_{D}$ is the hydrogen chemical potential, T is the temperature, and $f(E,\mu_{D},T)$ is the occupation function. N(E) is derived by partial differentiation of Equation (1). For $E=\mu_{D}$ the partial derivative ∂f/∂μ peaks. This allows to approximate the hydrogen density‐of‐states by [29]

(2) $$N_{D}\left(\mu_{D}\right)\approx \frac{\partial C_{D}}{\partial \mu_{D}}$$

**Figure 6:**
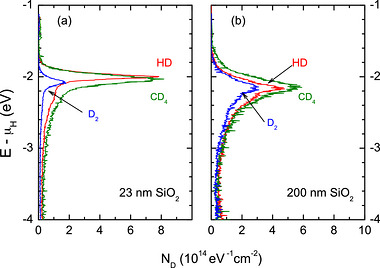
Hydrogen density‐of‐states, $N_{D}$, in single‐layer graphene transferred to c‐Si coated with (a) 23 nm and (b) 200 nm thick ${\mathrm{SiO}}_{2}$. $N_{D}$ was determined from the effusion data for $D_{2}$, HD, and ${\mathrm{CD}}_{4}$ shown in Figure 3.

In addition to the hydrogen concentration Equation (2) requires knowledge of the hydrogen chemical‐potential. This can be obtained from the molecular flux of the effusion measurement according to [29]

(3) $$\frac{dN}{dt}=N_{0}\times \text{exp}\left(-\frac{E-\mu_{D}}{k_{B}T}\right)$$

where the prefactor is given by $N_{0}=\nu N_{surf}$ for hydrogen desorption from the 2‐D graphene layer. The number of surface states is *N*_*surf*_ = 3.8 × 10^15^cm^−2^ and $\nu =10^{13}s^{-1}$ is the attempt frequency. Thus, the hydrogen density‐of‐states distribution can be calculated from the effusion spectra shown in Figure 3. $N_{D}$ is shown in Figure 6a,b for graphene transferred to substrates with a 23 and 200 nm thick ${\mathrm{SiO}}_{2}$, respectively. The data show that in graphene deuterium atoms are accommodated with a well defined binding energy that is influenced by the thickness of the oxide layer.

**Figure 7:**
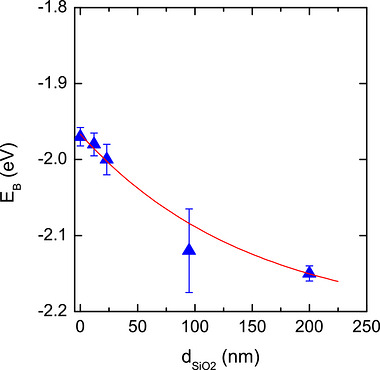
Effective hydrogen binding energy, $E_{B}$, as a function of the ${\mathrm{SiO}}_{2}$ thickness, 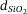.

The influence of the oxide layer thickness on the effective hydrogen binding‐energy, $E_{B}$, is shown in Figure 7. When graphene is transferred to oxide free (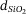 = 0 nm) c‐Si the effective H binding‐energy amounts to $E_{B}$ = 1.97 eV. With increasing oxide layer thickness, the effective H binding‐energy increases and reaches a value of $E_{B}$ = 2.15 eV for 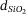 = 200 nm. From a thermodynamic point of view, one would expect $E_{B}$ to be independent of the oxide thickness. Since this is not the case the derived values for $E_{B}$ have to be interpreted as an effective binding energy that comprises potential kinetic barriers introduced by the substrate and the thermodynamic binding energy of deuterium/hydrogen.

The out‐diffusion of deuterium in an effusion experiment and the observation of $D_{2}$, HD, and ${\mathrm{CD}}_{4}$ requires the dissociation of C−D bonds. There are two possible paths for this mechanism: (i) the C−D bonds could dissociate when the D atom moves along the direction of the C−D bond or (ii) through an in‐plane wagging motion toward adjacent C−C bonds. For both reaction paths an energy barrier must be overcome. An estimate of the barrier height can be obtained by calculating the local vibrational modes. For the stretching and wagging vibrational modes frequencies of ω= 3138 and 1284 $cm^{-1}$ are obtained, respectively. This suggests that the energetic barrier for dissociation via the stretching motion (path (i)) is higher than for wagging motion (path (ii)). Hence, it is very likely that the dissociation of C−D occurs via path (ii).

**Figure 8:**
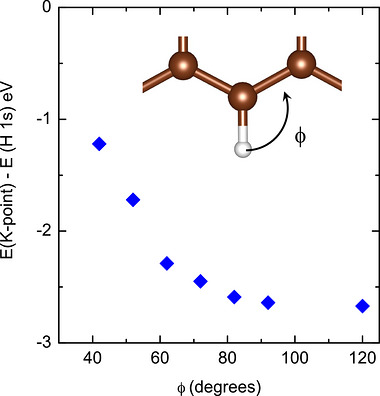
Distance of the hydrogen 1s orbital of an isolated C‐H complex to the Dirac point as a function of the bending angle, ϕ.

Assuming that the C−D bond length is constant during the wagging motion the position of the hydrogen s‐orbital was calculated as a function of the bending angle, ϕ. In Figure 8, the energy of the s‐orbital with respect to the Dirac point (E(K)) is shown as a function of ϕ. As the H atom moves closer to the adjacent C−C bond, an s‐like state moves closer to the Dirac point by about 1.45 eV for ϕ = 42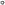 (see Figure 8). Interestingly, the corresponding anti‐bonding orbital (not shown here) is hardly affected by the change in bonding angle. It moves slightly away from the Dirac point from 4.84 at ϕ = 120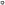 to 5.0 eV at ϕ = 42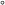. Dissociation of C−D can occur either by adding an electron to the anti‐bonding orbital or by removing an electron from the bonding orbital. Since the anti‐bonding orbital is well above the Dirac point and only the bonding orbital shows a pronounced decrease in energy as ϕ changes, it is very likely that the dissociation of C−D occurs via the capture of a hole in the bonding orbital. This mechanism is similar to minority carrier induced dissociation of hydrogen bonds in the bulk and on the surface of crystalline silicon [30, 31]. However, from a theoretical point of view, it differs in the behavior of the anti‐bonding orbital [31].

It is important to note that for the dissociation of all C−H bonds, a hole concentration of only about 6.9$\times 10^{13}$ is required. Most likely, the holes required for the dissociation of C−D bonds originate from the c‐Si substrate, where they are generated by thermal excitation during the effusion measurement. When an oxide layer is introduced, the holes can either tunnel or migrate through defects and pinholes in the oxide into the graphene layer. For thin oxides tunneling processes play an important role. However, when the 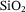 layer gets thicker charge carriers migrate through structural and/or electronic defect sites. Hence, for a thicker oxide, it takes longer for the charge carriers to reach the graphene layer. As a result, the peaks in the effusion spectra shift to higher temperatures, which manifests itself in an increase of the effective hydrogen binding energy, $E_{B}$.

## 6 Summary

In summary, hydrogen bonding in large area graphene was investigated. Gas effusion measurements show that out‐diffusion of hydrogen commences at a temperature of about 470 K. The effusion spectra show a single peak for the out‐diffusion of hydrogen that shifts toward higher temperatures with increasing thickness of the ${\mathrm{SiO}}_{2}$ layer. Integration of the effusion spectra yields the H concentration, which amounted to 1.2 − 6.9$\times 10^{13}$ ${\mathrm{cm}}^{-2}$. From the effusion spectra the H density‐of‐states distribution was derived, which shows that hydrogen is accommodated with an effective binding energy of $E_{B}$ = 1.97 eV. Interestingly, $E_{B}$ also exhibits a shift toward larger values with increasing oxide layer thickness. Most likely this is due to holes that need to diffuse through the 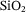 layer prior to being trapped in the bonding orbital of the C−H bond, which allows for spontaneous dissociation.

## Conflicts of Interest

The authors declares no conflicts of interest.

## Data Availability

The data that support the findings of this study are available from the corresponding author upon reasonable request.
